# Admixtures in Cement-Matrix Composites for Mechanical Reinforcement, Sustainability, and Smart Features

**DOI:** 10.3390/ma9120972

**Published:** 2016-11-30

**Authors:** Guillermo Bastos, Faustino Patiño-Barbeito, Faustino Patiño-Cambeiro, Julia Armesto

**Affiliations:** 1 Industrial Engineering School, University of Vigo, Rúa Conde de Torrecedeira 86, 36208 Vigo, Spain; inardesign.gbastos@uvigo.es; 2 Centro de Ciências Exatas e Tecnológicas, Centro Universitário Univates, Rua Avelino Tallini 171, Lajeado RS 95900-000, Brazil; faustino.cambeiro@univates.br; 3 Mining Engineering School, University of Vigo, Campus as Lagoas Marcosende, 36310 Vigo, Spain; julia@uvigo.es

**Keywords:** cement matrix, admixtures, reinforcement, multi-functional, recycled inclusions, alternative cements, sustainability

## Abstract

For more than a century, several inclusions have been mixed with Portland cement—nowadays the most-consumed construction material worldwide—to improve both the strength and durability required for construction. The present paper describes the different families of inclusions that can be combined with cement matrix and reviews the achievements reported to date regarding mechanical performance, as well as two other innovative functionalities of growing importance: reducing the high carbon footprint of Portland cement, and obtaining new smart features. Nanomaterials stand out in the production of such advanced features, allowing the construction of smart or multi-functional structures by means of thermal- and strain-sensing, and photocatalytic properties. The first self-cleaning concretes (photocatalytic) have reached the markets. In this sense, it is expected that smart concretes will be commercialized to address specialized needs in construction and architecture. Conversely, other inclusions that enhance strength or reduce the environmental impact remain in the research stage, in spite of the promising results reported in these issues. Despite the fact that such functionalities are especially profitable in the case of massive cement consumption, the shift from the deeply established Portland cement to green cements still has to overcome economic, institutional, and technical barriers.

## 1. Introduction

Concrete industry plays a significant role in global economy, to the extent that the consumption of its main component, Portland cement (PC), is highly dependent on GDP [1]. It is estimated that around 4.3 million tons of cement were consumed in 2015 worldwide, with a turnover of $335,000 million [2]. The Portland Cement Association [3] has predicted an annual 4% growth in this consumption from 2014 to 2016. Given such weight in economy, diverse research efforts are ongoing to improve both the fundamental properties required for construction materials, and other non-conventional properties. Likewise, several modifications are being tested in order to mitigate the high carbon footprint of PC.

Like mortar and cement paste, concrete is a cement matrix composite [4]. The cement matrix, which primarily determines the properties of this multi-phase material, consists in a three-dimensional lattice composed of hydrated cement phases [5] and, since the matrix is the continuous phase, it is the one that confers resistance to stresses. The properties of a composite derive both from those of its constituents and form their synergetic interactions. Therefore, most of the properties cannot be simply deduced from the rule of mixture, but should be identified by analyzing the physical interaction through their interfaces [6].

As structural composite material, the cement matrix consists of the combination of two or more non-miscible phases resulting in a macroscopically homogeneous material. In comparison with the matrix alone, after the addition of reinforcements, the interaction between the different components improves the properties of the matrix in terms of strength, weight, stiffness, fatigue, toughness, malleability, cracking resistance, etc. In the specific case of concrete, inclusions provide mechanical resistance in terms of surface hardness or maximum stresses, as shown in a stress–strain curve test [7]. In short, research is aimed at exploiting the synergies between the matrix and the inclusions that can improve the physical properties of the composite, through controlled dispersion and optimal proportion [8,9].

Given that cement has been used on a massive scale as construction material for a century, the different inclusions combined with the cement matrix have traditionally had the function of enhancing the structural performance of this composite. Several kinds of inclusions have either been already applied, are still in the research stage, or else have been adopted as future lines of research. The first toughening process made it possible to spread the use of PC in construction. It dates from the beginning of the 20th century and consisted initially in the use of iron reinforcing bars (rebars), currently substituted by corrugated steel bars. This technique provides reinforcement in the direction of the bars. In order to obtain additional strength in all directions, randomly distributed and finely dispersed elements are added to the mortar. Initially, natural fibers like asbestos were used [10]. In the second half of the 20th century, glass-fibers first, and next, alkali-resistant glass fibers [11], steel fibers [12,13,14], and synthetic fibers began to be added [15]. Nowadays, synthetic fibers dominate the market, representing nearly 47.87% of the total of the reinforcing fibers used in industry [16].

The scale of the reinforcing elements has progressively decreased as their effects at improving mechanical properties were observed. In this sense, micro-fibers, that present a greater specific surface area, were introduced. In the last decades, a set of supplementary cementitious micro-particles boosted the development of High Performance Concretes (HPC), being fly ash (FA), silica fume (SF), metakaolin (MK), and ground granulated blast-furnace slag (GGBS) the most used ones. Finally, more advanced nanomaterials have recently been introduced in the form of zero-, one- or two-dimension elements. Graphene-based materials reveal a high profile in terms of both their electrical properties and their good strengthening mechanism: their effectiveness at inhibiting the generation of micro-cracks is much higher than that of macroscopic aggregates, whose stress-absorbing properties takes place mainly after the appearance of micro cracks.

Apart from their classification depending on their mechanical performance, the most used families of inclusions can be described in terms of two new emerging functionalities, where achievements have been recently obtained: materials and manufacturing methods aimed at mitigating the high carbon footprint of PC; and the introduction of advanced new properties to the cement matrix. These new properties are obtained by using nanoparticles and carbon-based materials primarily thanks to their high reactivity, effectiveness as pore-fillers, and electrical properties, among other non-conventional characteristics.

In the last years, several reviews have been published with the goal of collecting and describing the most important findings and applications for several purposes of cement-based composites. Nanotechnology is a trend in this field. New reviews covering the fundamental aspects of nanomaterials—synthesis, mechanical reinforcement, and smart features—are being published [17,18,19,20]. Regarding environmental impact, other works have described energy-efficiency measures, as well as technologies that help to reduce the carbon footprint of Portland cement [21,22]. In this line, some reviews have presented recycled elements suitable to be incorporated to the ordinary cement matrix [23,24], and green alternative cements [25]. The existing literature on the subject of enhancement the mechanical performance of cement composites is usually presented in a fragmented way, referring specifically to certain reinforcements studied, such as fibers [26], nanomaterials [27], or pozzolanic materials [28]. In the present work, the authors bring together the studies that have dealt with the subject of the different types of inclusions used in cement composites in order to provide the aforementioned features. On one hand, this overview serves to compare the use of different inclusions in order to obtain a certain objective. On the other hand, it outlines the possibilities of combining different inclusions in order to provide different functionalities for cement composites, or to provide certain properties on a more efficient way.

The present work is structured as follows. Section 2 describes the different types of fibers, most of them commonly used as reinforcement addressed to absorb tensile stresses. Section 3 is dedicated to pozzolanic micro-particles, used to produce high-performance concrete since the 1970s. The significant environmental impact of cement production is assessed in Section 4: a number of recycled materials and alternative green cementitious materials are being investigated in order to mitigate this drawback of PC. Section 5 shows the interesting advances that nanomaterials bring, especially the graphene-based ones, both regarding mechanical performance and at providing new features and materials. The outcomes of the studies analyzed are discussed in Section 6, and the conclusions are presented in Section 7.

## 2. Fibers

The term “structural fibers” often refers to macro-fibers in lengths ranging from 19 to 60 mm and a diameter greater than 1 mm. Micro-fibers fall within 2–10 mm with a diameter of 0.1–1 mm [29,30]. Fibers have little effect on compressive strength. Their potential benefits can be observed in tensile strength and toughness, and are a consequence of two mechanisms. On the one hand, they absorb part of the load. However, their main contribution to strengthen the matrix is due to their crack- and pore-bridging capability [31], i.e., they do not retard cracking remarkably, but resist crack propagation and make failures more ductile. Fibers replace large single cracks with dense systems of micro-cracks. The stronger the fibers are, the more ductile becomes the failure.

A synergetic mechanical behavior is derived from the fiber/matrix contact. Fibers resist normal or tangential displacement of crack lips initiated in the matrix by transferring stresses to either side of such cracks [32]. Many factors affect the bond strength between fibers and cement matrix, such as roughness, aspect ratio, material, and fibrillation. The sensibility of such influence varies depending on the type of fiber [33].

With a high dosage of fibers, it is possible to obtain a cementitious composite that exhibits a strain-hardening behavior associated with the formation and propagation of cracks. If the fibers that are bridging a crack subjected to tensile stress have enough adherence to the matrix, and their tensile strength is greater than matrix strength, then, they can transfer load enough to induce new cracks [34]. In the case of pure tensile load, the result would be a series of subparallel cracks with an approximately equal crack spacing. This phenomenon, first introduced by Li and co-workers [35] is usually referred to as “pseudo strain-hardening”, due to the similarity between this behavior and the behavior of some steels [36].

**Figure 1:**
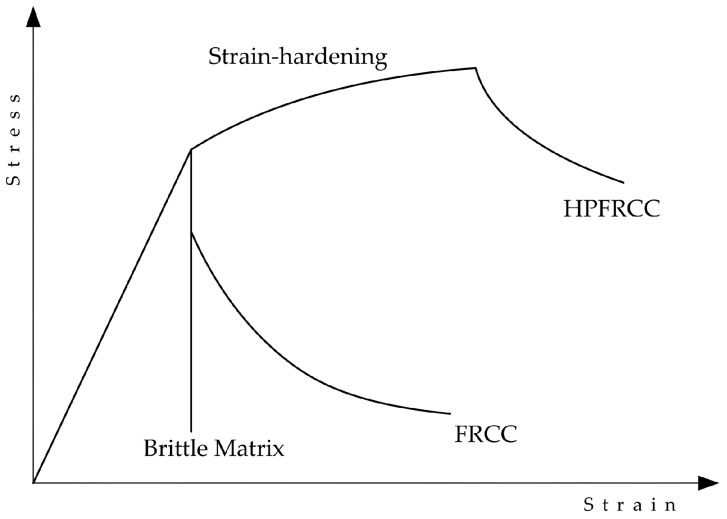
Failure behavior of plain cement matrix and fiber-reinforced cement matrices.

The fiber-reinforced cement composite (FRCC) that exhibits strain-hardening is commonly defined as High Performance Fiber-Reinforced Cementitious Composite (HPFRCC). The materials of the fibers used can be steel or polymeric [37]. Li and co-workers have introduced the concept of Engineered Cement Composite (ECC) as well. This cement composite is categorized under HPFRCC. A distinctive factor of ECCs lies on their microstructure, which is optimized using micromechanical models to achieve ultra-high ductility, limiting the crack width to below 100 µm and increasing ultimate tensile strain capacity up to 5% [38,39]. Figure 1 illustrates the enhanced resistance of HPFRCC compared to ordinary fiber-reinforced cement matrix.

Cracks can be caused not only by loads, but can be induced by the shrinking alkali-silica reaction in pore water, by the corrosion of rebars if present, or by the shrinkage occurred in curing [40]. The enhancement obtained in the strength of the cement matrix is proportional to the tensile strength of the fibers, their aspect ratio (i.e., the relation length/diameter) and the roughness of their surface [41]. The typical mechanical properties of the most common fibers used in cement matrix have been collected in Table 1.

### 2.1. Steel Fibers

**Figure 2:**
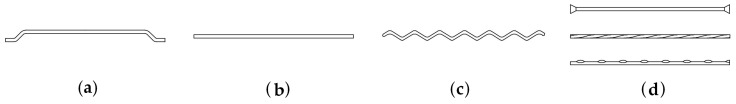
Most common steel fibers: (**a**) Hooked; (**b**) Straight; (**c**) Crimped; and (**d**) Other types.

Ever since they were introduced by Proter in 1910, steel fibers underwent no great development until the boost given by the publication of two papers by Romualdi and Batson in 1963 [12,13,48]. In the 1990s, steel fibers with a twist, with a higher pullout resistance, were introduced. In the 2000s, smooth and deformed ultra-high strength steel fibers appeared, with a minimum diameter of 0.12 mm and tensile strength up to 3400 MPa. A statistical analysis on the global market carried out by Katzer in 2006 [49] concluded that 67.1% of the steel fibers used worldwide belonged to the hooked type; 9.1% to the straight type; 7.9% to the crimped type, and 6.6% to other types. These types are shown in Figure 2. Steel fiber reinforced cement matrix is often used in pavements, thin shells and precast products, as well as in various patches and overlays [41].

Steel fibers constitute a solution to reduce the risk of shrinkage cracking, primarily in flat elements such as walls, slabs, and pavements. More generally, when an element is loaded until failure, the pullout of the fibers progresses gradually, and this provides ductility to the cracking, that otherwise would fail in a brittle manner. This ductility is achieved under all modes of loading, but the effectiveness in reinforcing varies depending on the type of loading, e.g., compression, flexural, shear and torsion stresses [50,51].

Once the exposed fibers corrode, such corrosion does not usually propagate much more than 2.5 mm deep. Some examples of the effects of fibers in cement matrices are shown in Table 2. They can vary substantially depending on the different materials used, their proportion, the type and elaboration method of specimens, and the testing devices and conditions.

### 2.2. Glass Fibers

The use of glass fibers in cement matrix was first attempted in the USSR in the 1950s [59]. It was quickly observed that ordinary borisilicate glass fibers (E-glass) are eroded due to the alkalinity of the cement paste. The corrosion of the fibers in the cement matrix causes a phenomenon commonly known as ageing of glass fibers in the cement matrix: the ductility and the enhancement of the mechanical properties of the composite decrease with time [60,61]. Considerable effort was made to develop alkali-resistant glass fibers, until this problem was practically solved by the addition of zircon oxide ZrO_2_ developed by Majumdar and Ryder in 1968 [11]. In the following years, new manufacturers introduced their alkali-resistant glass fibers (AR-glass).

Glass fibers are still not common in structural members, but their presence in facing and cladding panels and permanent formworks and shotcrete is relevant [62]. They provide a good response under fire, a relevant requirement for construction. Panels and other precast cement composites reinforced with glass fibers can be much thinner—typically 10 mm—than those reinforced with steel bars, which require a minimum cover of 30 mm as a protection against corrosion, reaching a similar strength. This is achieved not only thanks to the strength of glass fibers, but also to the lack of coarse aggregate, and a better water tightness, both of which allow a lower water/cement ratio [63,64].

A more specialized application of glass fibers takes place in the restoration of historical buildings. Due to the several drawbacks the common practice of using cement mortars in restoration works in terms of durability and chemical damage, the importance of the use of lime-based mortars is increasing. Lime-based mortars present better protective features: transpiration, dehumidifying capability and insulation. Iucolano et al. [65] highlight this fact. Focusing on cultural heritage buildings, they have stressed the potential of glass fibers mixed with lime mortar: specimens with 2% glass fibers were proven to increase compressive and flexural strengths by 80% and 66.2%, respectively, after 28 days of curing. A recent review of synthetic and natural fiber reinforced lime-based mortar was elaborated by Ramamurthi et al. [66].

### 2.3. Synthetic Fibers

The tensile strength of polymers is too low to serve as a main structural reinforcement under high loads. Synthetic fibers are used to limit the plastic shrinkage during the curing process, being often used as complement to another reinforcing inclusion. However, synthetic fibers provide noticeable improvements regarding compression and impact resistance [67]. A good example in this sense can be found in the experiments made by Richardson et al. [68], who concluded that the mechanical performance of concrete beams reinforced with a suitable dosage of polymeric fibers is comparable to that of steel fibers. The polymeric fibers used in their experiments consisted of 90% polypropylene (PP) and 10% polyethylene, with a 0.62 MPa tensile strength and dimensions of 40 × 1.67 × 0.095 mm^3^. In pullout tests, an equal performance was achieved with 40 kg/m^3^ of steel fiber specimens and 6.88 kg/m^3^ of synthetic fiber specimens.

PP fibers constitute a mature technology. They are the lightest of all synthetic fibers, and have a lower cost in comparison to other fibers, which is by far their main advantage. PP fibers are chemically inert and, therefore, corrosion will always affect the cement matrix before these fibers [69]. The most important reasons to use PP fibers in the cement matrix composites are their high effectiveness in the reduction of plastic shrinkage during hydration, and the improvement in toughness and energy absorption provided by them [70]. The enhancement in the compressive and flexural strengths obtained with PP fibers is still under study, especially in combination with other materials, such as pozzolanic microparticles [71], natural fibers [72], recycled admixtures [73], nanomaterials [74], or with fibers stronger than PP [75]. In contrast, the chemical composition of polypropylene makes the fibers hydrophobic in aqueous cement matrix, reducing the bond strength and hindering a uniform dispersion. A comparison of different surface treatments was made by Bodnárová et al. in order to tackle this weakness [76].

Polyvinyl alcohol (PVA) fibers can be used when higher reinforcement is required, for example in the case of the aforementioned ECCs. These fibers are surface-treated to optimize their dispersion within the cement matrix [46]. This treatment, together with the hydrophilic nature of PVA, results in a strong bond with the cement matrix [77]. Pakravan et al. [78] have compared the influence of PVA and PP fibers on flexural response of cement composites. They have observed an improvement in flexural strength by 10% and 48%, when adding 2% PVA fibers and 2% PP fibers, respectively. However, an important objective of their study is to highlight the partial replacement of PVA fibers by PP fibers as a way of reducing cost of FRCCs.

Cracks can occur in any stage of the life of concrete structures, commonly due to overload and corrosion. With the goal of solving the decrease in the performance and the reduction of the service life of concrete structures, different techniques have been developed in order to provide the self-filling capability to cement-based composites. Synthetic fibers play an interesting role in the achievement of this property, commonly known as autogenous healing [79].

An essential condition to obtain an autogenous healing is to control the crack width to a length below 150 µm and preferably below 50 µm. ECCs meet this requirement, as aforementioned. Their tight crack width is an intrinsic material property, as it is independent of loading, structure geometry, and steel rebars ratio [80]. A tight crack width is required to enable the healing process by means of the transport phenomenon. More specifically, the crystallization of calcium carbonate is the main mechanism involved in crack filling [81,82,83]. This reaction is stimulated by the presence of moisture, as in the case of wet/dry cycles in adverse environments [79], or freeze/thaw cycles [84]. Synthetic fibers can act as nucleation surfaces for those filling compounds, especially on bridging fibers [85,86]. Studies comparing the performance of the autogenous healing provided by synthetic fibers, report that PVA fibers facilitate the precipitation of quantities of healing-CaCO_3_ larger than those obtained with PP fibers [84,87].

Since water is required to enable the precipitation of CaCO_3_, superabsorbent polymers (SAPs) can be used to supply additional moisture to the unhydrated cementitious materials. SAPs are cross-linked polymers which can absorb water in huge amounts, up to 500 times their own weight [88]. Snoeck et al. [89] have studied the self-healing feature in mortar containing different proportions of PVA fibers and SAP particles. They have confirmed that the combination of both admixtures promotes the self-sealing of cracks, as well as the improvement in strength and durability associated to this structural healing. The same research group confirmed the self-sealing effect in mortar achieved by adding SAPs, with no fibers [90].

SAPs are also employed to compensate the shrinkage of concrete. This is especially useful in the case of HPCs due to their low water-to-cement ratio (w/c). With regard to the mechanical properties of the hardened cement composite, SAPs in combination with PVA fibers have been reported to initially diminish strength in early ages of the mixture and to improve it from approximately day 28 on [91]. Water-to-cement ratio must be limited in order to not to affect the strength. In this sense, Snoek et al. [88] have observed a light decrease in compressive strength of ordinary Portland mortar.

### 2.4. Carbon Fibers

In the last decades, the decrease in the cost of carbon fibers (CFs) and the continuous demand of superior structural properties and new functionalities has contributed to the introduction of carbon fibers as admixture in cement matrix. Short fibers with a typical length of 5 mm are less expensive. However, due to the weak bond between cement and CF, continuous CFs are much more effective. Surface treatment of the fibers has proved to be useful to counter this weakness [4].

CFs have piezoresistive characteristics: their electrical resistivity reversibly decreases and increases upon compressive and tensile deformation, respectively. Although piezoresistive carbon nanofibers and nanotubes—more novel materials—represent one of the trending research topics nowadays, the research of macroscopic CFs intended to obtain a piezoresistive behavior is still ongoing and yielding new results, primarily due to their lower cost. Moreover, the addition of very low carbon fiber contents is enough to provide this electrical feature to cement-based elements. Yeh et al. [92] noticed a good accuracy and sensitivity using a 0.2 vol % of carbon fibers, useful for detecting both elastic and plastic deformations.

In the context of real applications, Liu et al. [93] have highlighted the potential savings in the maintenance of asphalt concrete pavements achieved by providing self-structuring monitoring capacity using carbon fibers and graphite. Three stages of output resistivity were identified after lab tests. They correspond, respectively, to the contact tightening between mixture particles under the first phase of load, the smooth deformation of the concrete, and the generation and propagation of cracks in the failure phase. Savings in maintenance would be based on early repair works that would restore the pavement performance index up to 90%–100%, while restorations after manual inspection typically yield a restoration up to 30%–40%.

## 3. Pozzolanic Admixtures: High Performance Concrete

Pozzolanic materials are also known as supplementary cementitious materials because, if finely divided and in presence of moisture, they react chemically with the calcium hydroxide released by PC, contributing to the generation of the main component of the cement matrix: the calcium–silicate–hydrates gel (C–S–H) [5,94]. Another reinforcing effect consists in their pore-filler function. Cement paste is composed of small grains of the C–S–H, capillary pores (whose diameter falls in the order of the microns), nano-sized individual pores, and large crystals of hydrated products. There is room, therefore, for micro-sized pozzolanic particles to fill such pores and to make the cement product more compact [95]. A packed structure is essential to ensure a high durability of the cement matrix, since corroding agents progress more easily through irregularities with a high specific surface and through cavities, as it happens with the propagation of cracks that initiate the failure of the element.

Since the 1970s, pozzolanic admixtures have become necessary for the production of high strength concrete. In the 1980s and 1990s, the compressive strength of this type of concrete was increased from 80 to 120 MPa [96]. 

The concept of high strength concrete was soon replaced by that of High Performance Concrete (HPC), implying that additional characteristics had been enhanced: a lower heat of hydration allowed a lower thermal shrinkage, lower permeability provided a higher durability. Lower permeability also reduced the risk of attack by alkali-aggregates, sulfate soils and seawater, and also improved workability and reduced costs [97,98]. 

However, the secret of HPC does not lie on pozzolanic microparticles, but on the reduction of water content. The water-to-cement ratio is proportional to the distance between cement particles. The bigger the gap between cement particles, the more must the hydrates grow until they meet other hydrates, and create physical bonds. HPC is based on the use of high-range water reducers, named superplasticisers, which greatly increase the fluidity in the first stage of the blend. Ordinary concrete is mixed with a w/c usually from 0.42 to 0.60 and yields typically 40 MPa of compressive strength. HPC is produced with a w/c usually from 0.30 to 0.40, and from 0.25 to 0.30 in particular cases [99].

HPC is used in the construction of massive structures subjected to high loads, for example in tall buildings, large span bridges and dams. Some of the most used pozzolanic admixtures are described below: fly ash, silica fume, metakaolin and ground granulated blast-furnace slag.

### 3.1. Fly Ash

**Figure 3:**
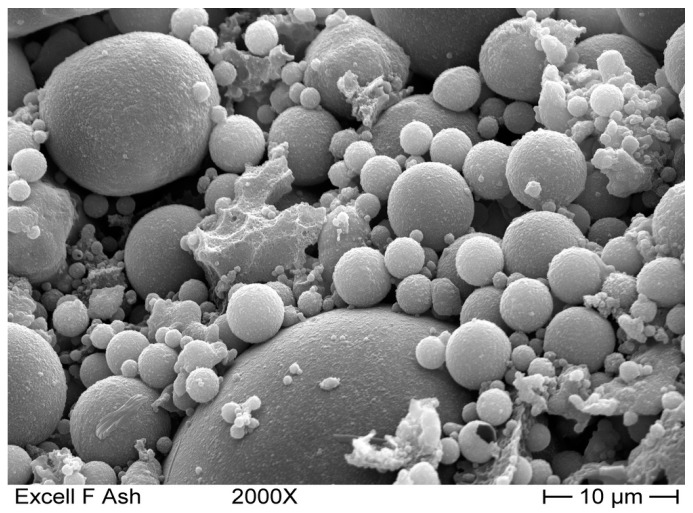
Magnified image of FA particles (reproduced with permission of Petra Buildcare Products [102], http://www.indiacenosphere.com/hollowSphere.html).

The most widely used pozzolanic material all over the world is FA, the finely divided residue from the combustion of powdered coal. It consists of spheroidal particles in a size range of 1 to 100 microns [100,101]. A sample of these particles is shown in Figure 3.

FA contributes to the strength development to a lesser extent than PC by itself. Since the pozzolanic reaction is slower than cement hydration, the initial compression strength of FA cement matrix is lower than the strength of ordinary cement. However, at a later age, the strength of the FA cement product may exceed that of the ordinary cement [94]. Due to the pore-filler function and the use of water reducers, a mortar with 10% FA can reach a 20% increase in the compressive strength compared to the ordinary cement matrix [103].

Apart from good technical characteristics, the massive adding of FA to cement matrix in construction industry also contributes to control the environmental impact [94]. FA is a waste product that must be landfilled if not used in other applications as a raw material in asphalt concrete or filling materials in embankments, harbors, etc. Since this by-product is used as a replacement for typically 50% of PC, it reduces the environmental impact of cement production [104,105].

As cement-based composites, the performance of HPCs depends on many components and parameters. Table 3 outlines the main characteristics of HPCs: the content of pozzolanic admixture and water, as well as the compressive resistance, the most searched property in these materials. It can be observed how the strength of the matrices with a high content of pozzolanic materials increases substantially over time.

### 3.2. Silica Fume

SF is a by-product obtained in the reduction of high purity quartz with coal in an electric arc furnace in the manufacture of silicon. It is composed primarily of pure silica (SiO_2_), usually over 92% [109]. Like FA, it has a spherical shape, but it is smaller in size: the average diameter of the particles is roughly 0.1 micron, about 100 times smaller than average cement particles. Its high specific surface area, together with an increase in the density of the mortar, makes necessary to add a superplasticiser to the mix [94]. SF improves compressive strength and abrasion resistance, and reduces permeability, not only because of the compaction function, water reduction and pozzolanic reaction (chemical formation of C–S–H), but also due to the bond strengthening caused by the thickening of the transition phase between the cement aggregates and the reduction the orientation of C–H crystals [109]. 

Modern high-strength cement matrices are based on SF mixed with superplasticisers. This mixture allows increasing the compressive strength of ordinary cement matrix from 40 to roughly 80 MPa [94]. This improvement is applied in HPC for highway bridges, parking decks, marine structures and bridge deck overlays, as well as in a variety of cementitious repair products, and also for rock stabilization in the form of shotcrete [109].

### 3.3. Metakaolin

Thermally activated ordinary clay (or kaolinitic clay) is a natural pozzolan. After purifying with a water treatment, a 100% reactive pozzolan known as metakaolin (MK) or highly reactive metakaolin results [94].

Apart from the strengthening and packing effects, MK makes the cement matrix lighter, increases the resistance to chemical attack, reduces the effects of alkali-silica reactivity, enhances workability, and reduces the risk of efflorescence [110]. Moreover, MK can be used alone or in combination with other less reactive cementing materials to produce high performance concrete without drawbacks such as an undesirable color or low workability [100].

### 3.4. Ground Granulated Blast-Furnace Slag

GGBS, or slag cement, is produced by grinding granulated blast-furnace slag, a waste product obtained during the manufacture of iron. GGBS is, in fact, a self-cementing material, as it is composed of silicate and aluminate [5]. It has been used in concrete production as a common supplementary cementing material for more than a century. It consists of particles not much finer than cement particles, more than 80% particles passing 45 µm sieve [100,111,112].

GGBS and FA have traditionally been treated as replacements for PC, while SF and MK tend to be applied as performance-enhancing additives. Although theoretically 100% of PC can be replaced by FA, replacement rates above 80% often require a chemical activator. In the case of GGBS, the optimal replacement from the strengthening point of view lies between 70% and 80% [113]. GGBS and FA are often the first option for producing high-strength cement matrix, reaching compressive strengths of at least 70 MPa. For higher values, typically above 80 MPa, supplementary portions of SF or MK can be quite useful [97].

## 4. Environmentally-Driven Admixtures

Approximately 5%–6% of global CO_2_ emissions originate from the manufacturing of PC, the most used kind of cement by far [114,115]. Therefore, PC contributes significantly to the climate change. With the growing awareness of environmental sustainability, modifications on PC production specifically addressed to deal with this problem are under research. Salas et al. [22] have reviewed the advances in its manufacturing process in four lines of action: energy efficiency measures, the reduction of fuel energy, PC replacements, and carbon capture storage. Supino et al. [116] have outlined the co-processing practice—i.e., the use of alternative materials and fuels—in Europe, with a focus on Italy and Germany. They have highlighted the European case as an example to be followed by the emerging economies. Mehta [117] reminds that simply by replacing the commonly adopted 28-day curing period by a 56-day one in standards, the cement consumption would be substantially reduced. 

In the context of inclusions, the main subject of the present paper, an increasingly important option is the replacement of PC with materials with a lower environmental impact. Such materials can be recycled inclusions whose disposal would cause a negative environmental impact, or manufactured materials that possess cementitious characteristics produced at a lower environmental cost.

Some industrial processes generate waste whose recycling is economically unsustainable due to the costs in logistics and treatment process. A usually desired alternative consists in converting such waste materials into inputs in another processes or products, shaping a circular production chain that minimizes the net inputs and outputs. Cement-based composites have already yielded positive results in this area, including admixtures usually sized from micro- to macro-scale. The construction sector itself is an important consumer of waste concrete aggregates. After reviewing the use of coarse recycled concrete aggregates, McNeil et al. [118] conclude that they are suitable for structural purposes, given that the decrease in mechanical properties is admissible: they show a comparable splitting tensile strength, but a higher deflection. Similar conclusions have been obtained by Correia et al. [119] in the case of ceramic waste, with the difference that they provide a higher abrasion resistance at the cost of a higher water absorption, and therefore less durability.

As it was shown in Section 3, the pozzolanic materials FA, SF and GGBS are by-products from the metal industry that have been necessary for the production of HPC for many years. Waste glass can be used as alternative pozzolanic recycled admixture thanks to its high content of silica. In the EU, circa nine Million tons of waste glass are generated annually [120], while 11.5 Mt were reported in USA in 2011 [121], most of which were not recycled but landfilled because they consisted of coloured glass, which would result in undesirable products [23]. Cement matrix with glass aggregates has the drawback of causing expansive reactions, but this problem decreases with the size of glass particles. Glass powder has been found to enhance mechanical properties both at microscale (75 µm) [122] and at nanoscale [123].

The disposal of waste tires is an issue of growing concern worldwide. Tires are composed of rubber and steel fibers. Recycled rubber can improve the performance of concrete, not only regarding mechanical properties. In the review made by Thomas et al. [124], the following common conclusions were found in literature when rubber content increases: compressive strength would be greatly affected, flexural strength is decreased with crumbed rubber or rubber powder and it is increased with rubber fibers, rubberized concrete is more resistant to freeze-thaw test and acid attack, but water absorption increases. Moreover, the increase in ductility and sound absorption makes rubberized concrete suitable to be used in traffic barriers and acoustic panels [125]. Steel fibers from waste tires enhance mechanical properties at a similar level of industrial steel fibers [126].

In a free market, the most economical materials are preferred among all that fulfill certain requirements. In order to promote the use of recycled admixtures, Governments can develop incentives and disincentives around traditional and recycled materials. However, a more efficient approach lies on identifying and highlighting the tangible and non-tangible benefits that recycling can bring to cement products [113].

Regarding green cementitious materials suitable to replace PC, interest has been renewed in MgO-cement and geopolymer cements as subjects of study. MgO-based cements used to be commonly used before the widespread of PC. The possibility of achieving a negative carbon footprint, their excellent seal effect because of a little expansion during curing, and the admission of a greater content of recycled materials in comparison to ordinary cement thanks to its lower alkalinity are examples of the advantages of commercial magnesium-based cements [21].

Geopolymer cements constitute a more intense research field. They are based on minimally processed natural materials and low-energy production processes. Some of their advantages are the abundance of the natural resources needed for its production, a low shrinkage, reasonably good hardening kinetics, an outstanding durability and a resistance maintained up to 1000–1200 °C [127]. FA- and GGBS-based cements are included in this family of products. Laboratory testing has shown a good potential for geopolymer concrete at a large scale, but practical applications are still not common. For instance, Wilkinson et al. [128] have highlighted the promising results in geopolymer cement matrix applied to pavements, but they have observed a lack of homologated standards: geopolymer concrete is currently produced, placed, cured and tested using ordinary cement standards.

Since PC has been firmly established as a construction for more than a century, it is difficult for alternative materials to penetrate the market. McLellan et al. [129] have found in the Australian market that costs of geopolymer cements range from 7% lower to 39% higher than PC depending on the availability of resources and transportation distances. Moreover, institutional and technical barriers must be overcome. Heidrich et al. [130] have underlined the following obstacles regarding the introduction of green cements: the development of specific standards, the provision of raw materials for their use depending on local availability, and a spreading of the research work focused on technical performance and long-term durability. Heidrich et al. have studied the Australian case, but other researchers [131] stress the need of standardization and the identification of technical properties, as well as the importance of the economic obstacles. In their description of the obstacles for more sustainable cements in the global context, Dewald et al. [132] have highlighted that public funding is more difficult to attract due to the widespread perception of PC as a low “low-tech” material that does not need to be further developed. They remind as well that, in the construction field, a decade is required from lab experiments until the commercialization of innovative products. 

Dry-mix (or ready-mixed) cement constitutes an interesting way of reducing gas emissions in the manufacturing process of PC. In its manufacturing, all ingredients are mixed in the factory, and consequently the addition of water to the mix is on required only on site. Dry-mix cement has a lower carbon footprint, since the production of aggregates and additives is centralized. Huang et al. [133] have estimated a 10% decrease in CO_2_ emissions by using dry-mix mortar in the interior and exterior plastering of a typical building in Taiwan. Furthermore, they point out that most companies avoid the use of dry-mix cement due to its higher cost, despite the fact that the plaster layer required with dry-mix mortar is 40% thinner.

## 5. Nanoinclusions

Advances in the manipulation of matter at the molecular level have enabled the development of composites containing nanoinclusions. In cement matrix, there are two fundamental types of nanomaterials: carbon-based and non-carbon-based ones. Most of the non-carbon-based nanomaterials consist in particles already known for a long time. A common effect in the cement matrix is usually an increase in compressive strength and durability because of the more efficient filling of the pores. Moreover, the reactivity of these zero-dimensional elements increases as their size decreases, and thus their chemical bonding with the cement matrix is enhanced.

The researched carbon-based nanomaterials combined with cement matrix are, in fact, graphene-based elements: graphite nanoplatelets (GNPs), carbon nanotubes (CNTs) and graphene oxide (GO). On the one hand, their extremely high specific surface area and the chemical treatments over their surface make them very efficient at absorbing tensile stresses. On the other hand, the exceptional electrical properties of CNTs and, to a lesser extent, of GNPs, open the possibility of producing “smart” structures. One of the most attractive advanced properties for researchers is the possibility of using the whole cement matrix structure as strain- or thermal sensor.

As it can be appreciated in the following sections, nanoinclusions are able to substantially increase the value of cement matrices by enhancing certain characteristics. However, there is no noticeable translation of nano-modified cements into commercial products. The main reason why nanomaterials are not being adopted in construction is the cost involved. Cement-based products are needed in massive amounts, and therefore small differences in their price per kilogram increase significantly the total cost of building projects [134].

A common characteristic of cement matrix composites improved with nanomaterials lies in the scattered nature of the measured mechanical properties. This is caused by the variability in the current technology to synthesize the materials, the methods of dispersion, the wide range of ingredients and proportions adopted by researchers, and the specimen preparations and tests. In view of this situation, a commitment to introduce standard definitions, methods, and quality levels around nanotechnology would help substantially to the promotion of the advances described in this section.

### 5.1. Nanoparticles

In the last decade, the modification of the cement matrix with nanoparticles has become a trend in research. As a result, a wide variety of properties can be obtained through the inclusion of nano-scaled compounds into the cement matrix. Some of the most commonly tested materials that have been shown to improve certain properties of the cement matrix are the pozzolanic nanosilica (SiO_2_), the metallic oxides nano-Fe_2_O_3_, nanotitania (TiO_2_), nanoalumina (Al_2_O_3_), and nano-MgO, and the salt nano-CaCO_3_. The use of nanoparticles implies a better packed structure and, consequently, higher compressive strength and corrosion resistance, lower shrinkage and permeability, and a longer life span of cement products [135,136].

The reduced size of these inclusions makes it convenient to apply certain techniques that disperse them uniformly in the aqueous cement blend. The most common methods are ultra-sonication and the use of superplasticisers. Ultra-sonication consists in transmitting excitation energy through a probe in order to apply external forces that exceed the internal attraction forces upon nanoparticles [18]. The use of superplasticisers is usually preferred and it does not require specific equipment. Superplasticisers are substances that cover the particles with a thin film and make them more soluble in aqueous environments. Therefore, the aforementioned fluidification effect that enables the production of HPC can be used to virtually increase the solubility of nanoparticles. Shaikh et al. [137] carried out a comparison of the compressive strength obtained in cement pastes with nanoparticles combining the use of different superplasticizers and the ultra-sonication method. 

**Figure 4:**
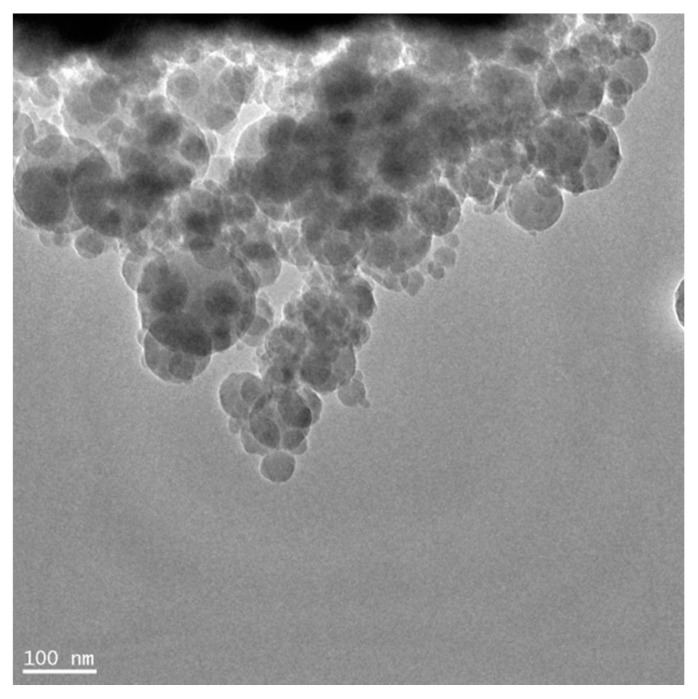
Transmission Electron Microscope (TEM) micrograph of nanosilica (reproduced from [143]).

The evolution in the manufacture of nanosilica has turned it into the most cost-effective nanoparticle, and the most investigated for its use in cement matrices. A sample of nanosilica can be seen in Figure 4. The experiments carried out by Zhang et al. [138] show the potential of the reinforcement of nanosilica: with 2% w/c of this particles, a 48.7% and 16.0% improvement in compressive and flexural strengths was achieved, respectively, in ordinary mortar. Zhang et al. dispersed the particles with the ultra-sonication method. Most of researchers have selected the use of superplasticizers with nanosilica [95,139]. The addition of pozzolanic FA [140]; SF [141]; and steel, polypropylene and glass fibers [142] to both elements has also been researched.

The interest in nanoparticles has led to experiments that combined different materials. Jalal et al. [144] have enhanced the strength and corrosion resistance of high-performance, self-compacting concrete by means of an efficient pore filling using micro- and nanosilica particles. A denser structure has been obtained by Jinchang et al. [145] in an UHPC by the combination of nanosilica and nano-CaCO_3_. The same two nanoparticles have been studied by Wu et al. [146], not only with the aim of strengthening Ultra-High Strength Concrete, but also to study the flowability, heat of hydration, phase change, and pore structure in such concrete. Li et al. [147] have presented an overview of improvements in compressive and flexural strengths of mortars containing nanosilica, nano-Fe_2_O_3_, SF, and combinations of them. Ismael et al. [148] reported an increase of roughly 25% of the bond between matrix and steel rebars using separately nanosilica and nanoalumina.

Beyond the reinforcing function, nanoparticles can modify some of the many several characteristics during the process that takes place from the pouring of the cement matrix mixes until the long-term hardening. As it was pointed in the case of the environmentally-driven MgO-cement, magnesium oxide provides a moderate expansion behavior to the cement matrix. Nano-MgO has been successfully used as an inclusion to deal with the shrinkage of the cement matrix during the curing [149]. The kinetics of cement hydration and thus its hardening can be altered by the addition of nanoparticles. Nano-CaCO_3_ can be applied as an accelerator (and additionally at lower shrinkage) of the hardening development [150], while nanoalumina can be used as a retardation additive [151].

Apart from nanosilica, the next most used nanoparticle is nanotitania [152]. TiO_2_ is a photocatalytic material: when added to cement matrix, a photocatalytic construction material is obtained. This material gathers solar radiation, which can decompose organic pollutants that come into contact with the nanoparticle [153]. It has been tested both as an admixture added to the cement blend [154] and as a coating, given that this phenomenon occurs on the surface of the cement product [155]. Jafari et al. [156] have found a better performance of the hybrid nano-TiO_2_-SiO_2_ coating compared to nano-TiO_2_ coating.

Another particles whose performance increases as their size decreases are lead zirconate titanate (PZT) particles. They provide piezoelectric behavior to the cement matrix, and they have been studied in the two last decades [157]. The piezoelectric behavior of a material consists in a change of the voltage across the material when it is subjected to a stress or load [158]. In this sense, the use of PZT-cement composites as sensors aimed at in situ real-time structural health monitoring is being investigated [159,160].

The piezoelectric properties of cement-based composites containing pozzolanic materials had been seldom investigated until Chaipanich et al. published their study on cement composites with PZT and SF [161]. Pan et al. [162] have tested cement paste containing PZT particles and GGBS, one of the pozzolanic materials that increase the strength as the composite ages. Pan et al. have studied how the age of the PZT cement composite affects its electrical behavior. They have found that most electric and piezoelectric properties become steady from the 60th day after polarization on.

Most of the research literature regarding these smart composites analyses how the piezoelectric and dielectric properties are influenced by the volume fraction and particle size of PZT [161]. Nanotechnology contributes to further advances in this line of research. For instance, Li et al. [163] have confirmed the superior piezoelectric performance of nano-PZT powder in comparison to coarse PZT particles.

Gong et al. [164] have reported that the piezoelectric sensitivity of the PZT cement can be substantially improved by incorporating small amounts of carbon black. This is an amorphous carbon-based material that does not contribute to the strength of the hardened composite, but provides electrical conductivity. Regarding recycled elements, Wang et al. [165] have reported that even PZT-cement mortar accepts the incorporation of waste materials such as LCD glass powder, while maintaining the piezoelectric feature.

### 5.2. Carbon-Based Nanoinclusions

Carbon has an unusually complex behavior at the molecular scale. Its great bonding capability with itself and with other elements constitutes the basis of organic compounds. In the framework of cement matrix composites, graphite nanoplatelets (GNPs), carbon nanotubes (CNTs) and graphene oxide (GO) have been under study in the last decades. All of them are, in fact, graphene-based materials: considering their molecular structure, they consist in different configurations of graphene nanosheets. Ironically, graphene was the last one of these elements to be isolated by Geim and Novoselov in 2007 [166]. It is composed of a one-atom thick planar net of carbon atoms bound to each other through hybrid sp2 bonds, resulting in a honeycomb structure [167]. Iijima was the first to present the carbon nanotubes in 1991. They are basically cylinders of graphene sheets [168]. Graphene oxide sheets are composed of graphene sheets bonded with carboxyl, hydroxyl and epoxy groups [169]. They have been known for more than a century and can be produced by exfoliating graphite flakes [170].

Graphene is approximately 130 times stronger than steel and outperforms copper in heat and electrical conductivities [171,172]. Pristine graphene nanosheets are not suitable for cement matrix because of the low solubility of graphene. Given that graphite is composed of panels of bonded graphene sheets, it inherits the characteristics of graphene partially, and it is used in the form of GNPs in reinforcing the cement matrix. Nevertheless, GNPs are still usually dispersed by chemical surface treatments. Zohhadi et al. [173], for instance, have studied the increase of flexural strength and stiffness of the cement paste with GNPs treated with surfactants to avoid the aggrupation of GNPs in aggregates. Meng et al. [174] have used GNPs to increase the compressive strength and toughness of Ultra-High Performance Concrete.

**Figure 5:**
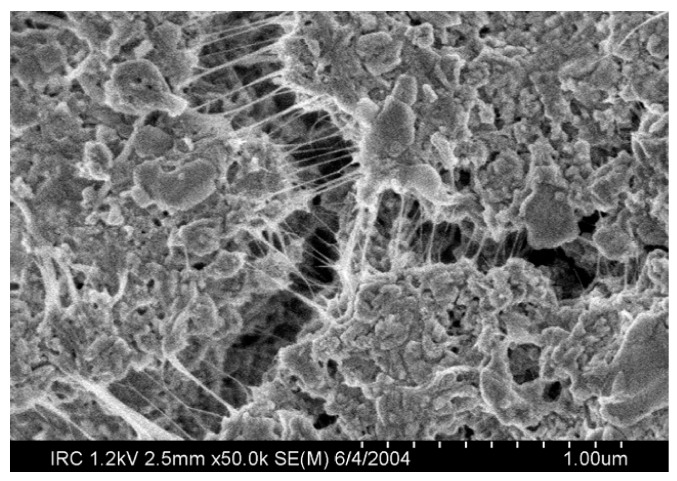
SEM micrograph. Crack bridging carried out by CNTs in OPC (reproduced from [135]).

CNTs and GO sheets can work as excellent reinforcing admixtures, since they avoid crack generation, unlike macroscopic fibers, which absorb stresses mainly once the failure has started. CNTs have a tensile strength of circa 100 times higher than that of the steel [175]. Both have been thoroughly tested in cement matrix, proving that they can compete with carbon fibers at reinforcing. While macroscopic fibers do not retard the failure of a structure noticeably, nanotubes avoid the generation of cracks more effectively (see Figure 5), apart from working as a pore filler [176]. A problem to be solved in order to produce cement matrix with CNTs is the uniformity of dispersion: due to their shape, their friction with the matrix is low and tends to form bundles. Superplasticiser and surfactants can be applied in this case [135]. The cement mortar tested by Alrekabi et al. [177] achieved greater compressive strength using surface treatments—both with superplasticiser first, and then with surfactant—compared to the strength obtained using functionalization. This dispersion method is a chemical treatment: carboxylic or hydroxyl groups are bonded to CNTs by the application of acid mixtures [178]. Chuah et al. [18] have produced an overview on the mechanical enhancement achieved by research groups using CNTs with different dispersion techniques.

The electrical properties of CNTs have been exploited in cement matrices in two main lines. One is based on the piezoresistive strain-sensing characteristic described in Section 2 regarding carbon fibers. Many studies have demonstrated the usefulness of these composites to monitor elastic and plastic strains [179,180]. The second can be observed in the study made by Zuo et al. [181]. They have highlighted their potential application as thermal-sensors in concrete structures due to the fact that electrical resistivity of cement matrices containing CNTs depends heavily on temperature. Interesting potential applications of thermal-sensing concrete structures are thermal monitoring of processes highly dependent on temperature, or in buildings and traffic structures where fire risk exists.

**Figure 6:**
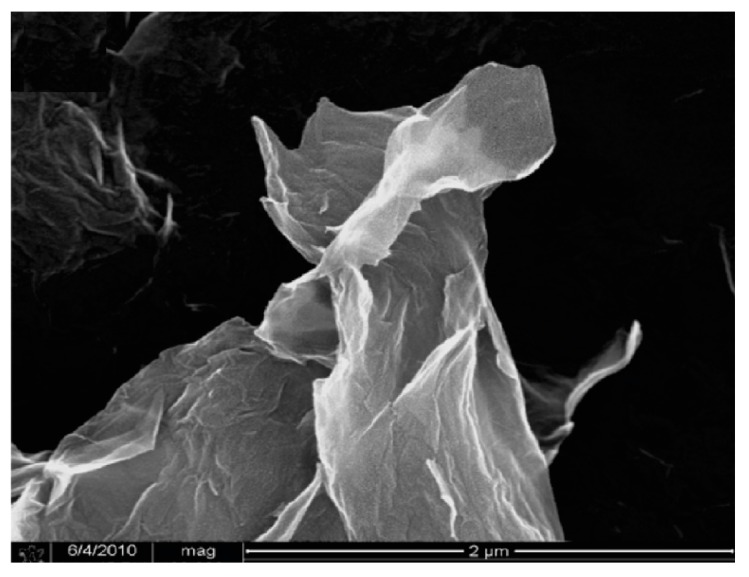
Scanning Electron Microscope (SEM) micrograph of GO (adapted with permission from [186]. Copyright © 2010 American Chemical Society).

GO has carboxyl, hydroxyl and epoxy functional groups that damage the valuable electrical properties of graphene, as well as its mechanical strength [169]. In turn, such molecules—which can be viewed as impurities in graphene—provide the GO with a high solubility in aqueous cement matrices. A micrograph of a GO sample can be seen in Figure 6. Moreover, GO is easier to produce at a high scale compared to pristine graphene and CNTs, and serves as a nucleation agent for C–S–H crystals [182]. For these reasons, the investigation of GO as a reinforcement of the cement matrix is gaining momentum [183,184]. For example, a low content of 0.05% w/c of GO dispersed with superplasticiser in the matrix resulted in an improvement by 24.4% and 70.5% in compressive and flexural strengths, respectively, in the tests carried out by Wang et al. [185].

Investigations on the combinations of these nanomaterials are ongoing. Li et al. [187] found a greater efficiency with the combination of GO and CNTs, obtaining a 72.7% increase in the flexural strength of the cement matrix, significantly higher than those of 51.2% obtained with GO and 26.3% with CNTs.

## 6. Discussion of Results

In view of the mechanical improvements provided by the different admixtures tested in cement matrix, it can be observed that, when a high compressive strength is desired, HPCs yield the best results due to the reduction in water content, along with their efficient pore-filling effect and the binder generation carried out by pozzolanic admixtures. In this sense, UHPCs constitute a state-of-the-art product, since they show a compressive strength over 150 MPa, which can reach up to 810 MPa in laboratory conditions [188]. HPC and UHPC enable the construction of more slender structures, increasing the living space in tall buildings, and thus allowing architects to design more complex forms by eliminating rebars [189]. Conversely, UHPC has certain weaknesses, such as a higher cost and thermal shrinkage, as well as a high environmental impact [190]. Shi et al. [191] have worked on a review of the materials currently used to produce these concretes, while the review by Wang et al. [183] describes their characteristics. As it has been pointed out in Section 2, fibers provide the possibility of controlling the shrinkage effect, and Yoo et al. [192] have used them to test an Ultra High Performance Fiber-Reinforced Concrete that allows a more satisfactory management of this problem.

As stated in Section 3, the pozzolanic micro-particles FA, SF, MK and GGBS have been used in the last decades to obtain a denser cement matrix. In the same direction, nanomaterials represent a further step, as they have proved to enter pores that are inaccessible for micro-particles. In order to fill pores at a lower cost, both micro-particles and nanoinclusions can be combined.

Regarding tensile strength and, by extension, flexural response, the improvement provided by admixtures is not enough to carry high loads, but admixtures can help to support steel rebars, or can even replace them completely if subjected to moderate loads. The aforementioned precast products containing steel or glass fibers, i.e., with no rebars, constitute an outstanding example in this sense. Such products enable the production of thinner structural shells or panels. HPFRCCs constitute another remarkable achievement in the field of fiber-based structural concretes. CNTs and GO increase the flexural resistance of cement matrices substantially, but these graphene-based nanomaterials imply expensive synthesizing methods and dispersion techniques in the cement blend, and, consequently, they remain in the research phase in the framework of cement matrices.

Fibers are not suitable to replace steel rebars completely in structural concrete. However, they greatly increase the toughness of the cement matrix, as it has been described in Section 2, and therefore energy absorption, which makes them useful in order to carry dynamic loads. By adding various types of fibers, cement matrices can resist dynamic loads—compressive, tensile and flexural—3–10 times greater than the loads resisted by cement paste [193]. PVA fibers allow, not only to produce ductile and strengthened concretes, but also to provide self-healing properties due to their capacity to fill cracks in the presence of moisture.

Most of the inclusions added to the cement matrix have the function of enhancing its strength and durability, allowing a reduction in cement consumption. Nevertheless, companies will not shift to more structurally efficient cements unless they become more economically attractive. Interesting results have been reported in alternative environmental practices. The most common one is the massive addition of recycled concrete aggregates to new cement-based products, a practice established long time ago. In many cases, waste products are not only added as a way of reducing the environmental impact of their disposal, but can also provide different properties to the composite. Low CO_2_ footprint cementitious materials, such as the aforementioned magnesium- and geopolymer-based cements, are gaining importance in the search of a more sustainable construction. Nevertheless, they are still rarely present in the market: Hasanbeigi et al. [21] place MgO-cement and geopolymers-cement at pilot and demonstration stage, respectively, and cement containing FA and recycled elements at a semi-commercial phase.

Both lines of action—recycled inclusions and low carbon footprint cements—converge in the emerging concept of “green concrete”. This concept refers to environmentally friendly concretes regarding the following key parameters: quantity of materials used as PC replacement, manufacture process, performance, and life cycle sustainability [194]. However, a more significant reduction in PC consumption must still be achieved in order to tackle long-term environmental problems, since global warming is causing damages both to human health [195] and economy [196].

The most innovative features developed in cement matrix are provided by nanoparticles and carbon-based materials. The reduced size of nanoparticles and their high reactivity make them suitable to act as strengthening admixtures. Experiments with GO and functionalized CNTs have shown good reinforcing features, due to the fact that these elements have a specific surface area extremely higher than that of the 0-D nanoparticles. Piezoresistive behavior of CNTs can provide strain- and thermal-sensing capability to cement-based structures, and this feature can be obtained using the lower-cost carbon fibers, or even graphite. Promising results have been obtained regarding a more economical maintenance of asphalt concrete [48], and the construction of traffic infrastructures made of concrete that act as anomalous temperatures or weight-in-motion sensors, vehicle flow measurers [181]. Such advanced concretes remain in the research stage. Piezoelectric behavior is a more complex and advanced characteristic than piezoresistivity, and it has been already tested to produce cement-based sensors that monitor structural health in real time.

Thanks to their high reactivity, nanomaterials have been proven to modify the properties and evolution of the fresh cement blend, mainly hardening its kinetics, heat of hydration, and flowability. What is more, certain properties that emerge at the nanoscale have already been applied in cement matrices. The photocatalytic features of TiO_2_ stand out among nanoparticles. Some buildings constructed with self-cleaning TiO_2_-concrete are presented in the study by Banerjee et al. [197] in connection to state-of-the-art self-cleaning technology based on TiO_2_, along with Italian and German companies that are offering currently TiO_2_-concrete.

In view of the range of inclusions that enhance the behavior of cement matrix in a variety of aspects, the combination of different types of admixtures has become a flourishing field of research, especially regarding the family of nanoparticles. This increases the complexity of identifying the nano-modified cement matrix composites most suitable to be translated into commercial products. 

## 7. Conclusions

The present work provides a review of the families of inclusions added to the cement matrix that are already being used or are currently under research, based on three different functions: the development of mechanical performance, a requirement for the high-scale use of cement in construction; alternative materials that reduce the high carbon footprint of traditional cement products; and the achievement of smart features that fulfill specific needs in construction. 

Given that most of cement-based products have a structural function, the mechanical properties of the cement matrix are still being pushed to higher levels with the application of the state-of-the-art materials described in the present paper. Firstly, the decrease in the size of admixtures provided by nanomaterials enables a higher pore-filling effect. Secondly, and from a chemical approach, nanomaterials can act as nucleation agents for cement matrix crystals, whereas chemical treatments of admixtures enhance their bond to the matrix. Thirdly, the combination of different types of admixtures constitutes a productive but complex field of research. Different mechanisms of reinforcement can work simultaneously by means of an adequate gradation of size: steel rebars, reinforcing fibers, pozzolanic micro-particles, and the different families of nanomaterials. 

Certain inclusions provide non-conventional features to cement-based structures. Such advanced features are not intended for an extensive application in cement products but for certain elements. Strain- and thermal-sensing features provided by carbon-based materials are expected to be applied in structures subjected to high loads, dynamic loads, or structures that involve the safety of users, such as buildings and traffic pathways. It is expected that advanced features will be exploited to develop highly valuable, commercial cement products, aimed at meeting special needs in construction. In this sense, the photocatalytic property of nanotitania has already been applied in architecture for the construction of low-maintenance-cleaning buildings.

In applications with a massive PC consumption, many obstacles emerge in the shifting from traditional cement to more developed materials. High-scale consumption makes the construction sector more sensitive to prices of materials and to transportation distances. However, evolution from ordinary to stronger or green cements requires an evolution in several non-economic aspects: the perception of companies and users regarding the value of green concretes, standards that support and facilitate the development of new technologies applied to cement, and optimized research resources. This demands the contribution of all the actors implied: funding, regulating and education support from public organizations, the promotion of quality and innovation through building certificates, and the commitment from business associations. The accomplishments in this field may bring not only efficiency in construction, but may mitigate the future impact of global warming on human health and economy.

## Figures and Tables

**Table 1:** Typical mechanical properties of fibers.

Fiber Type	Elastic Modulus (GPa)	Tensile Strength (Gpa)	Elongation at Break (%)	Diameter (µm)	Aspect Ratio	Source
Steel	200	1.5	3.2	500	20	[42]
Glass (E-glass)	72	3.45	4.8	5–10	600–1500	[18,43,44]
Glass (AR-glass)	70–76	1.8–3.5	2	12	600–1500	[18,45]
Polypropylene	8	0.1–0.8	8.1	100	150	[42,46]
Polyvinyl alcohol	29–36	0.8	5.7	14–650	430–860	[46,47]
Carbon	240	2.5	1.4	7	710	[42]

**Table 2:** Examples of improvement in strength (str.) by fibers in cement-based composites.

Volume Fraction of Fibers	Properties of Fiber	Type of Cement-based Mixture ^1^	Increase in Performance ^2^	Ref.
1.5% Steel fibers	Hooked type; 33 mm length; 0.55 mm diameter; 1.25 GPa tensile str.	Concrete; superplasticiser	87% split tensile str.; 72% flexural str.	[50]
1.0% Steel fibers	Hooked type; 60 mm length; 0.80 mm diameter; 0.66 GPa tensile str.	Concrete; superplasticiser	59.0% split tensile str. 68.7% flexural str.	[52]
0.1% Glass fibers	12 mm length; 14 µm diameter; 72 GPa elastic modulus;	Concrete; OPC 53 grade	42.23% split tensile str.; 19.31% flexural str.	[53]
1.5% Polypropylene fibers	15 mm length; 100 µm diameter; 0.4 GPa tensile str.	Concrete; superplasticiser	−35.6% 1st crack str. −33.3% flexural str.; 27.3% toughness (I_5_)	[54]
0.75% Polypropylene fibers	12 mm length;	Pavement concrete; OPC 53 grade	11.9% compressive str.; 9.7% flexural str.	[55]
0.25% PVA fibers	6 mm length; 14 µm diameter; 1.5 GPa tensile str.	Concrete; Shrinkage limited PC; Fly ash	11.7% compressive str.; 32.4% split tensile str.; 21.4% flexural str.	[56]
2% Carbon fibers	3 mm length; 26.8 µm diameter; 0.86 GPa tensile str.	Mortar	140% tensile str.; 187% flexural str.	[57]
0.8% Carbon fibers	3 mm length; 7 µm diameter; 3.45 GPa tensile str.	Mortar; Superplasticiser; Carbon fibers with hydrophilic surface modification; Dispersed through ultrasonication	17.9% flexural str.	[58]

^1^ Unless otherwise specified, ordinary PC ASTM Type I was used; ^2^ Specimens cured for 28 days.

**Table 3:** Examples of mechanical properties of HPCs.

Pozzolanic Admixture and Weight of Cement	Water-to-Cement Ratio/Water-to-Binder Ratio	Compressive Str. at the 28th Day (MPa)	Compressive Str. at the 56th Day (MPa)	Source
15% SF	0.27 w/c	101.0	103.5	[106]
5% MK	0.3 w/c	89.0	94.0	[106]
30% FA	0.41 w/b	72.5	90.3	[107]
33% GGBS	0.42 w/b	40.7	52.3	[108]
